# Public acceptance of cybernetic avatars in the service sector: evidence from a large-scale survey

**DOI:** 10.3389/frobt.2025.1719342

**Published:** 2026-01-12

**Authors:** Laura Aymerich-Franch, Tarek Taha, Takahiro Miyashita, Hiroko Kamide, Hiroshi Ishiguro, Paolo Dario

**Affiliations:** 1 Robotics Lab, Dubai Future Labs, Dubai Future Foundation, Dubai, United Arab Emirates; 2 Interaction Science Laboratories, Advanced Telecommunications Research Institute International, Kyoto, Japan; 3 Graduate School of Law, Kyoto University, Kyoto, Japan; 4 Department of Systems Innovation, The University of Osaka, Osaka, Japan; 5 The Biorobotics Institute, Scuola Superiore Sant’Anna, Pisa, Italy

**Keywords:** cybernetic avatars, human-robot interaction, multicultural contexts, robot avatars, social robots, technology acceptance

## Abstract

Cybernetic avatars are hybrid interaction robots or digital representations that combine autonomous capabilities with teleoperated control. This study investigates the acceptance of cybernetic avatars, with particular emphasis on robot avatars for customer service. Specifically, we explore how acceptance varies as a function of modality (physical vs. virtual), robot appearance (e.g., android, robotic-looking, cartoonish), deployment settings (e.g., shopping malls, hotels, hospitals), and functional tasks (e.g., providing information, patrolling). To this end, we conducted a large-scale survey with over 1,000 participants in Dubai. As one of the most multicultural societies worldwide, Dubai offers a rare opportunity to capture opinions from multiple cultural clusters within a single setting simultaneously, thereby overcoming the limitations of nationally bound samples and providing a more global picture of acceptance. Overall, cybernetic avatars received a high level of acceptance, with physical robot avatars receiving higher acceptance than digital avatars. In terms of appearance, robot avatars with a highly anthropomorphic robotic appearance were the most accepted, followed by cartoonish designs and androids. Animal-like appearances received the lowest level of acceptance. Among the tasks, providing information and guidance was rated as the most valued. Shopping malls, airports, public transport stations, and museums were the settings with the highest acceptance, whereas healthcare-related spaces received lower levels of support. An analysis by community cluster revealed, among other findings, that Emirati respondents were particularly accepting of android appearances, whereas participants from the ‘Other Asia’ cluster were particularly accepting of cartoonish appearances. Our study underscores the importance of incorporating citizen feedback from the early stages of design and deployment to enhance societal acceptance of cybernetic avatars.

## 1 Introduction

Cybernetic avatars are hybrid interaction robots or digital representations that can perform tasks on their own, while also being controlled by a human operator (Horikawa et al., 2023; Ishiguro, 2021; Ishiguro et al., 2025). Operators can interact socially through cybernetic avatars, control the avatar body, and communicate verbally through it (Aymerich-Franch et al., 2020; Dafarra et al., 2024; Hatada et al., 2024; Kishore et al., 2016). These entities are envisioned as a technology that can free humans from limitations of body, brain, space, and time (Ishiguro, 2021; Ishiguro et al., 2025). Robot avatars are primarily explored in real-world applications as surrogate bodies for individuals with reduced mobility (Hatada et al., 2024) and for remote travel and physical interaction, such as virtual museum visits (Roussou et al., 2001). Additionally, virtual avatars are extensively used for real-world applications in gaming and interaction in social virtual reality applications, improving mental health, and promoting behavioral change (Aymerich-Franch, 2020a; Aymerich-Franch et al., 2014; Falconer et al., 2016; Rosenberg et al., 2013; Slater et al., 2019; Yee and Bailenson, 2007).

Our study examines social acceptance of cybernetic avatars, with a particular emphasis on robot avatars for customer service. Acceptance is broadly conceptualized as intention to use, or actual use, of a given technology (Davis, 1989; Naneva et al., 2020; Venkatesh et al., 2003). More specifically, for the purposes of our study, acceptance is defined as the cybernetic avatar being willingly incorporated into the society (Broadbent et al., 2009).

Technology acceptance has been extensively examined in the literature, most notably through the Technology Acceptance Model (TAM), which identifies perceived usefulness and perceived ease of use as the primary determinants of acceptance (Davis, 1989), and the Unified Theory of Acceptance and Use of Technology (UTAUT), which highlights performance expectancy, effort expectancy, social influence, and facilitating conditions as key determinants of acceptance (Venkatesh et al., 2003).

In the particular domain of social robots, De Graaf and Ben Allouch (2013) identified perceived usefulness, adaptability, enjoyment, sociability, companionship, and perceived behavioral control as particularly relevant in relation to acceptance of social robots.

Other factors, including exposure to social robots, intended domain of application, the design of the robot, or demographic characteristics such as gender, age, and geographical location have also been analyzed as potential factors impacting acceptance of these robots (Gou et al., 2018). Among them, appearance has received considerable academic attention (Broadbent et al., 2009; Goetz et al., 2003; Kanda et al., 2008; Katz and Halpern, 2014; Li et al., 2010; Robins et al., 2004). Social robots are specifically designed for interaction, and therefore require certain social cues such as eyes or voice to support natural and effective communication with users. However, the well-known “uncanny valley” hypothesis (Mori et al., 2012), which proposes that people experience discomfort or eeriness when a robot appears almost, but not fully, human, has generated extensive debate and a large body of research on whether human-like designs could negatively influence acceptance (Destephe et al., 2015; Robins et al., 2004).

For social robots deployed in service sectors, situational factors also play a decisive role and need careful consideration. It is important to understand how the tasks robots are expected to perform, as well as the environments in which they operate, influence user acceptance. In this sense, prior survey research shows that acceptance can vary substantially depending on the nature of the task assigned to the robot and the type of space in which it is deployed. (Aymerich-Franch and Gómez, 2024; European Commission, 2012).

Finally, cultural differences have also received important consideration in the investigation of robot acceptance (Bartneck et al., 2007; Broadbent et al., 2009; Kaplan, 2004). A substantial body of work identifies cultural background as a significant determinant of perceptions, expectations, and acceptance of robots (Arai et al., 2019; Bartneck et al., 2007; Kamide et al., 2025; Kamide and Arai, 2017; Nomura et al., 2008). Cybernetic avatars have been progressively deployed in Japan. However, acceptance of this technology in other demographic contexts might differ (Kamide et al., 2025). In this regard, Dubai presents a particularly valuable case study to examine acceptance of cybernetic avatars in multicultural societies, as over 200 nationalities live and work in the UAE (UAE Ministry of Foreign Affairs, 2024). In fact, the Emirati population of Dubai is estimated to be about 8%, and 92% are non-Emirati (Dubai Statistics Center, 2024).

Additionally, Dubai’s demographically diverse environment provides a rare opportunity to examine the acceptance of cybernetic avatars across highly varied geographical backgrounds within a single urban setting. This study capitalizes on the Emirate’s cosmopolitan character to present a uniquely global perspective on the social acceptance of this emerging technology.

To gain a comprehensive understanding of cybernetic avatar acceptance, we conducted a large-scale survey involving over 1,000 participants. In particular, we explored how cybernetic avatar acceptance varies as a function of modality (physical robot vs. virtual), robot appearance (e.g., android, robotic-looking, cartoonish), deployment settings (e.g., shopping malls, hotels, hospitals), functional tasks (e.g., providing information, patrolling), as well as geographical origin (community clusters). To our knowledge, his constitutes the largest study to date investigating the acceptance of cybernetic avatars within such a culturally heterogeneous population.

### 1.1 Avatar appearance and modality

At present, cybernetic avatars present two primary modalities: physical robot avatars and virtual reality avatars (Aymerich-Franch, 2020b; Horikawa et al., 2023; Ishiguro et al., 2025). The first research question explored acceptance of the two modalities:

RQ1. To what extent are robot and virtual avatars accepted, among Dubai residents?

To address RQ1, survey participants were asked to envision an ideal society for Dubai and indicate on a three-point scale (disagree–neutral–agree) whether avatars designed to assist customers in the service sector would take the form of physical robots and/or virtual representations.

The second research question explored acceptance in relation to different robot avatar appearances:

RQ2. To what extent are different robot avatar appearances accepted, among Dubai residents?

**FIGURE 1:**
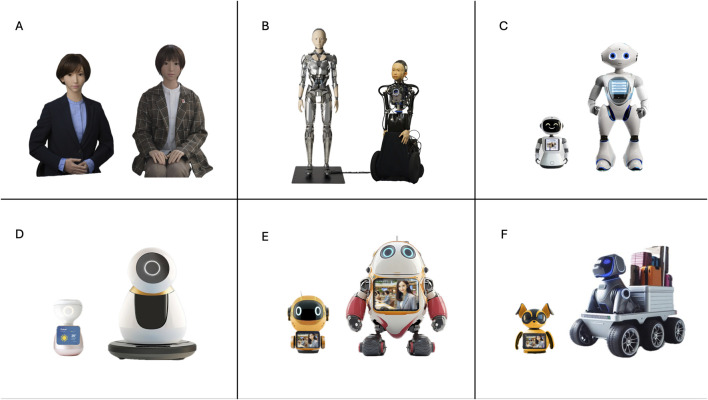
Robot avatar appearances assessed in the survey.

To explore RQ2, we developed a typology of six distinct categories that represent the primary social robot appearances currently being used in the service sector worldwide based on previous works (Aymerich-Franch and Ferrer, 2020; 2023; Glas et al., 2016; Nakata et al., 2022). The final typology included six groups: ultra-realistic android, hybrid android (human-robotic looking), highly anthropomorphic robotic looking, low anthropomorphic robotic looking, cartoonish looking, and animal looking. We then selected two visual representations which exemplified each category in the survey. For the two android categories, we borrowed images from Erica, Yamatoroid, and Ibuki, representing both adult and child android versions, which are androids designed by Ishiguro and colleagues (Glas et al., 2016; Nakata et al., 2022). For the remaining categories, we generated *ad hoc* designs with the aid of graphic designers and AI tools. Because social robots vary in size and mobility, we incorporated this variation into the *ad hoc* designs. For each category, one exemplar represented a smaller, static desktop robot, while the other represented a larger, mobile robot. This ensured that size and mobility features were not tied to any specific appearance category. Figure 1 shows the robot avatar pictures and designs that exemplified each category in the survey. Participants were asked to envision an ideal society for Dubai and indicate whether robot avatars designed to assist customers in the service sector would have each of the appearances.

### 1.2 Spaces in the service sector

The third research question explored robot avatar acceptance depending on the setting where robot avatars are found:

RQ3. To what extent are robot avatars accepted in the different spaces of the service sector, among Dubai residents?

We elaborated a list of key spaces in healthcare, education, financial, retail, transportation, hospitality, and government services where robot avatars could potentially be introduced, based on the environments where social robots for customer service are commonly deployed in real-world scenarios, as identified by Aymerich-Franch and Ferrer (2020), Aymerich-Franch and Ferrer (2022), and Aymerich-Franch and Ferrer (2023). Participants were asked whether, in the ideal future society they envisioned for Dubai, robot avatars would be permitted in these spaces (e.g., shopping malls, hospitals, schools).

### 1.3 Tasks for the service sector

We also explored robot avatar acceptance in Dubai depending on the task they perform:

RQ4. To what extent are robot avatars accepted to perform the different tasks typically performed by social robots, among Dubai residents?

For that, we elaborated a list of general tasks, drawing on the roles that social robots currently fulfill in real-world settings, as identified by Aymerich-Franch and Ferrer (2020), Aymerich-Franch and Ferrer (2022), and Aymerich-Franch and Ferrer (2023). The functions included providing information to customers, customer registration/check in–out, providing indications and guidance to find a place, telepresence to speak with the human controlling the robot, patrolling spaces for security, companionship and entertainment functions, object delivery, on-site customer data collection and feedback collection. We asked participants whether in the ideal future society they envisioned for Dubai, robot avatars would be used for these tasks in the service sector.

### 1.4 Relationship with robots and demographic variables

We additionally collected information related to relationship with robots. In particular, we included general level of interest in scientific discoveries and technological developments, general view of robots and attitudes towards robots’ scale (items based on Aymerich-Franch and Gómez (2024); European Commission (2012), and fear of robots’ scale (items based on Aymerich-Franch and Gómez, 2024; Liang and Lee, 2017).

Regarding demographics, we collected information on nationality, gender, age, occupation, time living in the UAE, born in the UAE, level of studies, income level, religious beliefs, background in Computer Science, level of programming skills, experience using robots, experience interacting with social robots and robot avatars, and collectivism–individualism orientation (Triandis and Gelfand, 1998).

## 2 Methodology

### 2.1 Contents of the survey

The survey contained the following blocks^1^:

- Introduction to cybernetic avatars
- Acceptance of robot avatars by appearance (ultra-realistic androids, hybrid androids, highly anthropomorphic robotic-looking, low anthropomorphic robotic looking, cartoonish looking, and animal looking)
- Acceptance of robot avatars by space (e.g., shopping malls, banks, etc.)
- Acceptance of robot avatars by task (e.g., provide information, patrolling, etc.)
- Acceptance of cybernetic avatars by modality (robotic and virtual)
- Relationship with robots and technology (interest in science and technology, attitudes towards robots, fear of robots)
- Demographics

We additionally added two attention check questions among the previous questions (e.g., “I am paying attention to the survey, select “XX”). A full list of questions is provided in the Supplementary Material (SI).

### 2.2 Platform

The survey was outsourced to an external market research firm, which was responsible for recruiting participants and administering the survey through its platform. The survey was conducted in English. Participants were compensated with points, which could be accumulated and exchanged for rewards. Data collection took place between December 2024 and February 2025. Upon completion of the survey, the market research firm provided the raw data in Excel format, which we used for subsequent analysis.

### 2.3 Participants

The survey was administered in English and it was open to participants aged 18 or older, residents of Dubai. The survey was initiated by 4,000 participants. Of these, 1,542 were screened out due to ineligibility, 883 were excluded after quota targets were reached, 198 did not finalize the survey, and 376 were removed through quality control checks. These measures helped ensure a high-quality and demographically balanced dataset for analysis. The final sample consisted of 1,001 participants.

#### 2.3.1 Community clusters distribution

Dubai presents an atypical demographic structure. The population size of the Emirate of Dubai is estimated at 3.8 million, having the highest population among the emirates in the UAE. Of them, 31.4% are females and 68.6% are males. The majority of the residents (58.49%) are aged between 25 and 44 years.

Over 200 nationalities live and work in the UAE (UAE Ministry of Foreign Affairs, 2024). Given that the objective of the study was to capture perspectives from all major communities residing in Dubai, rather than to obtain a statistically representative sample of the general population, we employed a structured sampling strategy. Specifically, a stratified sampling method was used, based on gender and community affiliation, to ensure balanced representation.

For that, we identified the expat communities with 10,000 residents or more in the UAE (Wikipedia, 2024). A total of 35 countries and areas were identified. We then classified the countries by geographical region following the classification provided by the Statistics Division of the United Nations for statistical use (UNSD, 2024) to get an understanding of the geographical distribution of the represented groups. The initial classification resulted in countries and areas in the following regions: Eastern, South-Eastern, Southern, and Western Asia; Northern and Sub-Saharan Africa; Eastern, Northern, Southern and Western Europe; Northern America; Australia and NZ. We then further organized the countries and regions into six main community clusters that best reflect Dubai’s social composition: Emiratis (UAE citizens), a Middle East cluster (neighboring countries), a South Asian cluster (representing the largest expatriate group, comprising nearly 60% of the UAE population), and three additional clusters for ‘Other Asian’, ‘Other African’, and ‘Western’.

## 3 Results

The final sample consisted of 1,001 valid participants, 503 females and 498 males. Participants were distributed across six community clusters, each comprising between 157 and 174 individuals, as shown in Table 1. Of the participants, 4.1% were between 18 and 24 years old, 38.0% were 25–34, 38.4% were 35–44, and 19.6% were 45 or older. Most respondents (42%) had lived in Dubai between 4 and 10 years, with 24.1% born in the UAE. Over half (53.6%) indicated a high interest in scientific discoveries and technological developments, and the majority expressed a positive general view of robots, with 53.7% fairly positive, and 31.3% very positive. Fear of robots was moderate overall: 30.9% reported no fear, while 53.2% reported slightly or moderate fear. Most participants had occasionally interacted with robots (58.2%), and 54.1% had occasionally interacted with avatars.

**TABLE 1:** Clusters by country/areas, and sample representation in the large-scale survey.

Cluster	Countries/Areas	Participants (N)
Emirati	United Arab Emirates	170 (83 males, 87 females)
Middle East	Egypt, Iraq, Jordan, Lebanon, Palestine, Syria, Saudi Arabia, Türkiye, Qatar, Kuwait, Bahrain, Oman	166 (84 males, 82 females)
Southern Asia	India, Pakistan, Bangladesh, Nepal, Sri Lanka	173 (89 males, 84 females)
Other Asia	Philippines, China, Indonesia, Japan, Taiwan, Malaysia, Myanmar, Singapore, Kazakhstan, Uzbekistan, Armenia, Azerbaijan	174 (81 males, 93 females)
Western	European Union (Austria, Bulgaria, Croatia, Cyprus, Finland, France, Germany, Greece, Ireland, Italy, Lithuania, Poland, Portugal, Romania, Sweden), United Kingdom and overseas territories, United States and territories, Russia, Canada, Australia, Switzerland, Belarus, Bosnia and Herzegovina, Ukraine, Serbia, New Zealand, Albania, Peru, Cuba, Panama, Dominican Republic	161 (85 males, 76 females)
Other Africa	Ethiopia, Kenya, Benin, Cameroon, Ghana, Central African Republic, Congo, Côte d’Ivoire, Eritrea, Gambia, Ghana, Guinea, Nigeria, Rwanda, Sierra Leone, Tanzania, Togo, Uganda, Zimbabwe, Comoros	157 (76 males, 81 females)

### 3.1 Cybernetic avatar acceptance by modality: digital vs. robotic

**FIGURE 2:**
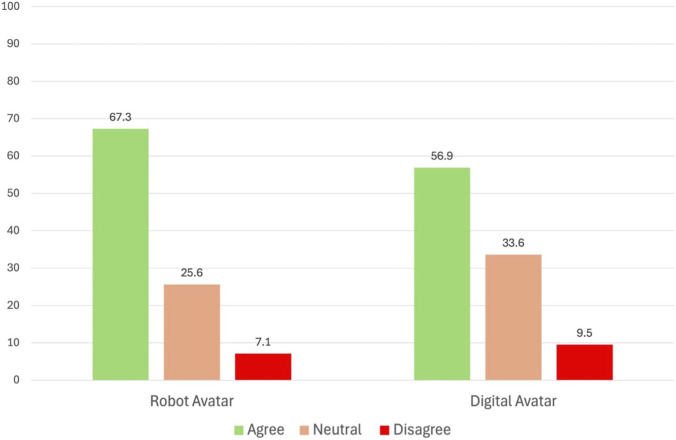
Acceptance rate by avatar modality: robotic and digital.

Overall, acceptance of cybernetic avatars was high. Acceptance of physical robots was higher than that of digital formats such as virtual avatars presented on screens or in VR environments (Figure 2). Specifically, 67.3% of respondents agreed with the deployment of robots in customer-facing service roles, compared to 56.9% who agreed with the use of digital avatars. A Wilcoxon Signed-Rank Test showed a significant preference for robots over digital formats (Z = −8.91, p < 0.001).

We then examined acceptance by nationality groups (community clusters). Acceptance of physical robots was consistently high across clusters, with 74.1% of Emirati respondents agreeing, followed by 70.5% acceptance from the Middle East cluster, 67.1% acceptance from the South Asia cluster, 65.0% acceptance from the Other Africa cluster, 63.8% acceptance from Other Asian countries, and 63.4% acceptance from the Western cluster. Acceptance of digital avatars was slightly lower. In particular, agreement was highest among Emirati (64.7%) and Other Africa cluster (62.4%) participants, followed by Westerners (60.9%), Other Asians (55.2%), Middle Easterners (51.2%), and South Asians (48.0%).

To examine differences in acceptance of physical and digital avatar formats across community clusters, we conducted two Chi-Square Tests of Independence, one for each modality. The three-level acceptance response (disagree, neutral, agree) was cross-tabulated with the six community clusters.

Acceptance of physical robot avatars differed significantly across community clusters, χ^2^ (10, N = 1,001) = 19.81, *p* = 0.031, although the effect size was small (Cramer’s V = 0.099). Examination of adjusted standardized residuals indicated that Emirati respondents agreed with the use of physical robots at significantly higher-than-expected rates (ASR = 2.1), indicating higher acceptance, and South Asian respondents were more likely than expected to select “Disagree” (ASR = 2.8), indicating higher rejection. No other clusters differed significantly from expected frequencies (Supplementary Tables S1A–I). Acceptance of digital avatars also differed significantly across community clusters, χ^2^ (10, N = 1,001) = 19.42, *p* = 0.035, with a small effect size (Cramer’s V = 0.098). Examination of adjusted standardized residuals also indicated that Emirati respondents showed significantly higher-than-expected agreement with the use of digital avatars (ASR = 2.2), indicating higher acceptance, and South Asian respondents showed significantly lower-than-expected agreement (ASR = −2.6) and significantly higher-than-expected disagreement (ASR = 2.7), indicating higher rejection and lower acceptance. No other clusters differed significantly from expected frequencies (Supplementary Tables S2A–I). Taken together, these findings suggest that Emiratis exhibit higher acceptance of both physical and digital avatar formats, while South Asians tend to be less accepting of both modalities.

A Mann-Whitney U test was conducted to assess gender differences in the acceptance of robot avatars. Although the median score was the same for men and women (Mdn = 3), the distribution of responses differed significantly (U = 113,829.00, z = −3.03, p = 0.002), with men showing greater overall acceptance. No significant gender differences (Mdn = 3) were observed for digital avatars (U = 123,512.00, z = −0.43, p = 0.667).

### 3.2 Cybernetic avatar acceptance by appearance

**FIGURE 3:**
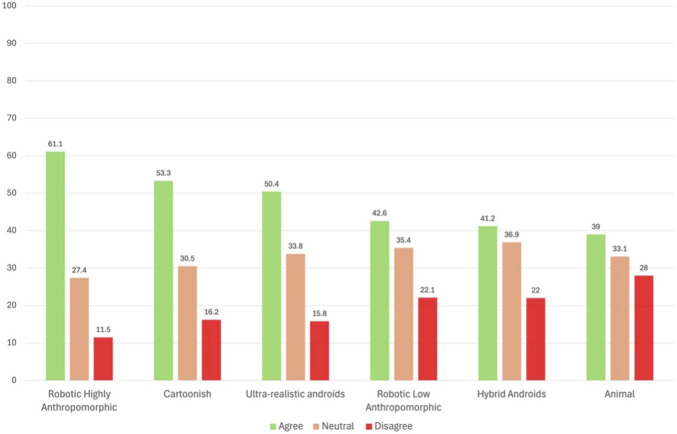
Acceptance rate by Appearance.

Overall, acceptance of robot avatars for customer service was very high, with the majority of respondents expressing agreement or neutrality across all appearance types. Robotic-looking avatars with high anthropomorphism received the highest level of agreement (61.1% agreed). This was followed by cartoonish (53.3%) and android designs (50.4%), both of which also received majority support. In contrast, robotic-looking avatars with low anthropomorphism (42.6%) and hybrid androids (41.2%) received more moderate support, potentially reflecting ambivalence toward designs that fall between clearly human or clearly robotic. Animal-looking robots were the least favored, with only 39% agreeing with their use in service roles, and the highest level of disagreement among all categories (28.0%), suggesting lower acceptability for zoomorphic forms in this context. Overall, the data suggest that the public in Dubai tends to prefer robot appearances that are distinctly anthropomorphic either in a robotic, human-like or cartoonish form, with less acceptance for ambiguous or zoomorphic designs (Figure 3).

#### 3.2.1 Appearance by community and gender

To examine whether acceptance of different robot avatar appearances varied across community clusters, we conducted a series of Chi-Square Tests of Independence, one for each of the six appearance types (android, hybrid android, highly anthropomorphic robotic-looking, low anthropomorphic robotic-looking, cartoonish, and animal-looking). For each appearance category, the three-level acceptance response (disagree, neutral, agree) was cross-tabulated with the six community clusters. Significant overall Chi-Square results were followed by an inspection of adjusted standardized residuals (ASRs) to identify which clusters showed higher- or lower-than-expected levels of agreement or disagreement with each appearance. Only ASRs with an absolute value of 2.0 or greater (approx. p < 0.05) were considered.

Acceptance of android robot appearances differed significantly across community clusters, χ^2^ (10, N = 1,001) = 59.11, *p* < 0.001, with a small-to-moderate effect size (Cramer’s V = 0.172). Examination of adjusted standardized residuals revealed that Emirati respondents showed much higher-than-expected agreement with android appearances (ASR = 5.4), and lower disagreement responses (ASR = −2.3), indicating stronger acceptance and lower rejection. Participants from the Other Asian cluster displayed significantly lower-than-expected agreement (ASR = −5.0), suggesting lower acceptance. Western respondents showed significantly higher disagreement with android appearances (ASR = 2.7), suggesting higher rejection. Regarding hybrid android robot appearances, acceptance also differed significantly across community clusters, χ^2^ (10, N = 1,001) = 19.47, p = 0.035, although the effect size was small (Cramer’s V = 0.099). Examination of adjusted standardized residuals revealed several meaningful deviations from expected response patterns. Emirati participants showed significantly fewer “Disagree” responses (ASR = −2.3), suggesting lower rejection of hybrid androids. Other Asian respondents displayed significantly fewer agreements (ASR = −2.8), indicating comparatively lower acceptance towards this appearance type. Western participants showed significantly more disagreement than expected (ASR = 2.0), reflecting higher rejection. Acceptance of highly anthropomorphic robotic-looking avatars did not differ significantly across community clusters. A Chi-square test of independence showed that the distribution of responses (disagree, neutral, agree) was comparable across groups, χ^2^ (10, N = 1,001) = 17.20, p = 0.070, with a small effect size (Cramer’s V = 0.093). Significant differences were also found in acceptance of low anthropomorphic robotic-looking avatars across community clusters, χ^2^ (10, N = 1,001) = 38.56, p < 0.001, with a small-to-moderate effect size (Cramer’s V = 0.139). Emirati respondents reported more agreement (ASR = 2.2) and fewer disagreement responses (ASR = −4.0) than expected, suggesting higher acceptance and lower rejection. In contrast, South Asian participants showed significantly lower acceptance of this appearance type, with fewer “Agree” responses (ASR = −2.3), while Other African respondents showed significantly higher disagreement (ASR = 2.4), suggesting higher rejection. Respondents from the Other Asian cluster showed fewer-than-expected disagreement responses (ASR = −3.3), indicating comparatively lower rejection. Acceptance of cartoonish robot also differed significantly across community clusters, χ^2^ (10, N = 1,001) = 40.70, p < 0.001, with a small-to-moderate effect size (Cramer’s V = 0.143). Adjusted standardized residuals revealed that participants from the Other Asian cluster showed substantially higher acceptance and lower rejection of cartoonish robots, with significantly more “Agree” responses (ASR = 4.4) and fewer “Disagree” responses (ASR = −4.1). In contrast, Western respondents reported more “Disagree” responses (ASR = 2.6) and fewer “Agree” responses (ASR = −3.6), suggesting higher rejection and lower acceptance. South Asian participants also expressed significantly higher disagreement responses (ASR = 2.3), suggesting higher rejection. Finally, significant differences in acceptance of animal-like robot appearances were also identified across community clusters, χ^2^ (10, N = 1,001) = 25.51, p = 0.004, with a small effect size (Cramer’s V = 0.113). Adjusted standardized residuals indicated that Emirati respondents showed significantly higher “Agree” responses for animal-like robots (ASR = 2.2), suggesting higher acceptance. Western respondents (−2.1) and Middle Eastern participants (ASR = −2) showed fewer-than-expected agreements, suggesting lower acceptance. The Other Asian cluster exhibited fewer-than-expected disagreements (ASR = −3.1). Differences in the neutral response category were also observed for several clusters and are additionally reported in the SI (Supplementary Tables S3A–7A).

In sum, the most pronounced differences in appearance preferences emerged for android and cartoonish robot designs. Within the android category, the strongest effects were observed among Emirati respondents, who showed notably higher acceptance, and participants from the ‘Other Asia’ cluster, who showed markedly lower acceptance compared to expected levels. Within the cartoonish category, the strongest effects were found for the ‘Other Asia’ respondents, who displayed a strong preference for cartoonish robot designs, and for Western participants, who showed much lower acceptance. Taken together, the results suggest that acceptance of different appearances varies significantly across community clusters as only acceptance of highly anthropomorphic robotic-looking avatars was comparable across clusters.

To assess whether acceptance of different robot avatar appearance types varied by gender, we conducted Mann–Whitney U tests comparing male and female participants’ responses across the six appearance types (Supplementary Tables S9A–I). Each response was treated as ordinal (Disagree = 1, Neutral = 2, Agree = 3). A Bonferroni correction was applied to account for multiple comparisons across the six appearance types (adjusted α = 0.0083). For the Android appearance, men (Mdn = 3) reported significantly higher acceptance (U = 108,832.50, z = −3.94, p < 0.001) than women (Mdn = 2). This difference remained statistically significant after Bonferroni correction. No significant gender differences were observed for the other appearance types, including Hybrid Android, Robotic High Anthropomorphic, Robotic Low Anthropomorphic, Cartoonish, or Animal-like avatars (ps > 0.05).

These findings suggest that acceptance of robot avatar appearances is meaningfully influenced by cultural identity and, to a lesser extent, by gender.

#### 3.2.2 Reasons behind android acceptance and rejection

Given that we were particularly interested in understanding the reasons behind acceptance or rejection of ultra-realistic androids, we included an additional open-ended question to further enquire about participant’s choice (agree–neutral–disagree). A thematic analysis was conducted on 663 open-ended responses from participants who either agreed (n = 505) or disagreed (n = 158) with the deployment of ultra-realistic androids in customer service roles. Neutral responses were excluded. Open-ended responses were initially analyzed using AI-assisted methods to identify recurring semantic patterns and identify key themes by grouping responses based on keyword similarity and latent linguistic features using lexical matching and semantic proximity. We then reviewed and refined the preliminary themes to obtain the final analysis.

Among those who agreed, four dominant themes emerged. The first theme, *Comfort and Familiarity*, reflected the perception that human-like robots made interactions feel more natural and less intimidating. For example, one participant stated, “I feel like it will be more comfortable to talk if its human like” (P24), while another noted, “it makes it more homely” (P149). The second theme, *Alignment with Dubai’s Innovation Agenda*, emphasized the congruence between human-like androids and Dubai’s identity as a technologically advanced, future-oriented city. One respondent remarked, “Dubai is a cosmopolitan city that embraces innovation and futuristic technologies. Deploying ultra-realistic androids for customer service aligns with this forward-thinking image and can attract tourists and residents” (P15). The third theme, *Enhanced Customer Experience and operational efficiency*, included references to service effectiveness enabled by realistic appearances (e.g., “To have a feeling of more realistic customer support”, P230; “They are fast and require less time to solve the issue. Financially beneficial over the humans. They can work 365 days without resting.”, P163). Finally, the fourth theme, *Public Appeal and Child Engagement*, highlighted the attractiveness of androids for diverse audiences, including children: “So that people can relate and not be scared of them especially children” (P654).

Among participants who disagreed, four opposing themes were identified. The first theme, *Uncanny Valley and Emotional Discomfort*, expressed unease with overly human-like robots, such as “Human like robots make me feel uncomfortable and a bit scary, I have a feeling that I am talking with a dead person” (P31) and “It’s more realistic and less creepy for Robots to look like machines rather than humans” (P40). The second theme, *Job Displacement*, emphasized concerns about automation reducing employment opportunities: “It takes jobs away from actual people who do these jobs now” (P43). The third theme, *Clear Human-Robot Distinction*, reflected the belief that robots should remain visually and functionally distinct from humans, as illustrated by “The robot should appear like a robot not similar to the humans” (P188) and “I will like robot to be distinctive and not to be confused with humans from far or near” (P53). The final theme, *Ethical, Moral, Philosophical, and Religious Concerns*, encompassed deeper ethical, moral, and philosophical reservations about the societal implications of anthropomorphizing machines: “I believe everyone is unique in his own way. I see no reason why the robot should look like a human, it should also have its own unique appearance. Also there will be a thin line between humans and ultra realistic androids that we should keep away from because we then loose the real sense of reality. We should take into consideration the younger generations to come should be able to identify and differentiate human from robots. I know having robots are part of the future everything changes so fast that if they look like us too it will confuse many people.” (P384), as well as religious: “God created humans and robots should not resemble us for that reason. And I feel better with a robot that does not have human features (P701) and “I have nothing against it, I just think it's not good to imitate humans. God honored humans, created things that resemble them, for me it's denigrating them.” (P695).

### 3.3 Spaces in the service sector

To address RQ3, regarding the extent to which robot avatars are accepted in the different spaces of the service sector among Dubai residents, participants were asked to rate their agreement with the deployment of robot avatars across 20 different settings. The results revealed considerable variation in acceptance depending on the type of space. Commercial and retail environments received the highest levels of approval, with 74.5% of respondents agreeing with robot deployment in shopping malls, followed by 61.8% in supermarkets, 60.2% in stores, and 59.6% in hotels. Similarly high acceptance was found in transport and transit hubs, with 69.6% agreement for airports and 68.9% for train and metro stations. Cultural and public leisure spaces also showed favorable attitudes, with 69.1% agreement in museums, 61.9% in libraries, and 58.1% in public parks and streets. In contrast, more moderate acceptance was reported for government and administrative services: 57.9% agreed with robot use in post offices, 49.6% in government offices, and 48.7% in banks. Attitudes were mixed in educational institutions, with 50.4% agreement for language academies, 49.7% for universities, and a notably lower 39.4% for schools and high schools. The lowest levels of acceptance were consistently found in healthcare and wellness settings. Only 37.4% of respondents agreed with robot avatars in hospitals and clinics, 35.9% in rehabilitation centers, 35.3% in pharmacies, 32.3% in nursing homes, and 31.6% in dental clinics. These results suggest that robot avatars are largely accepted in commercial, transport, and cultural spaces, where their roles are likely perceived as task-oriented and non-intrusive. Conversely, the hesitancy observed in government, educational, and especially healthcare settings may reflect concerns about trust, privacy, and the suitability of robotic agents in emotionally sensitive environments. Table 2 shows the full distribution of agreement, neutrality, and disagreement across all service settings included in the study.

**TABLE 2:** Acceptance of robot avatars across service sector settings (N = 1,001).

Setting	Agree (%)	Neutral (%)	Disagree (%)
Commercial and retail			
Shopping malls	74.5	16.3	9.2
Supermarkets	61.8	25.4	12.8
Stores	60.2	26.8	13.0
Hotels	59.6	26.4	14.0
Transport			
Airports	69.6	19.6	10.8
Train and metro stations	68.9	20.9	10.2
Cultural and public leisure			
Museums	69.1	20.2	10.7
Libraries	61.9	26.5	11.6
Public parks/streets	58.1	27.1	14.8
Government and administrative services			
Post offices	57.9	28.1	14.0
Government offices	49.6	30.6	19.8
Banks	48.7	30.1	21.2
Educational institutions			
Language academies	50.4	29.8	19.8
Universities	49.7	31.0	19.3
Schools and high schools	39.4	33.0	27.6
Event and professional venues			
Conference centers	53.5	30.2	16.3
Healthcare and wellness			
Hospitals and clinics	37.4	34.3	28.3
Rehabilitation centers	35.9	35.4	28.7
Pharmacies	35.3	34.5	30.2
Nursing homes	32.3	36.5	31.2
Dental clinics	31.6	33.1	35.3

### 3.4 Tasks for the service sector

Regarding RQ4, which asked to what extent robot avatars are accepted to perform the different tasks typically performed by social robots, the data revealed a generally favorable attitude among Dubai residents toward robot avatars performing a wide array of tasks commonly associated with social robots. The highest levels of acceptance were observed for utilitarian service-oriented tasks, such as providing information to customers (72.5% agreement), offering guidance to find a place (71.7%), and managing recycling collection (68.5%). Similarly, carrying personal items like shopping bags or suitcases (67.9%) and offering multilingual support (66.7%) were also widely accepted. Tasks related to procedural or administrative support, such as customer registration/check-in (66.0%) and collecting feedback (65.7%), received comparable endorsement.

However, more socially nuanced or emotionally sensitive roles yielded lower levels of acceptance. In particular, only 50.2% agreed that robot avatars could effectively provide companionship and entertainment, and even fewer respondents supported their use for handling customer complaints (43.2%), the lowest among all tasks. Intermediate acceptance was noted for object delivery (58.9%), loyalty program sign-ups (56.7%), security patrolling (55.6%), and telepresence-based service interactions (55.4%).

Overall, the findings suggest that while functional and informational roles for robot avatars enjoy broad support, tasks requiring empathy, discretion, or human rapport evoke more ambivalence or resistance among participants. Table 3 presents the percentage of respondents who agreed, disagreed, or remained neutral regarding the use of robot avatars to perform the different tasks.

**TABLE 3:** Acceptance of robot avatars for various social robot tasks among Dubai residents (N = 1,001).

Task	Agree (%)	Neutral (%)	Disagree (%)
Provide information to customers	72.5	18.1	9.4
Provide guidance to find a place	71.7	22.7	5.6
Recycling collection	68.5	21.1	10.4
Carry shopping bags or suitcase	67.9	21.8	10.3
Multilingual support	66.7	26.9	6.4
Customer registration/check-in – out	66	23.4	10.6
Customer feedback and surveys	65.7	24.6	9.7
Object delivery	58.9	28.1	13
Sign up to loyalty programs	56.7	30.8	12.5
Patrolling spaces for security	55.6	27.6	16.8
Telepresence with a service representative	55.4	27.1	17.5
Companionship and entertainment	50.2	32.2	17.6
Handle customer complaints	43.2	28	28.9

## 4 Discussion

This study examined public acceptance of cybernetic avatars—both physical robots and digital representations—in the multicultural environment of Dubai. The central goal was to understand how acceptance varies across key dimensions, including modality, visual appearance, deployment context, functional role, and demographic background. The research was motivated by the growing integration of cybernetic systems into public services and the need to align technological deployment with societal expectations in diverse communities.

Overall, the results reveal that acceptance of cybernetic avatars in Dubai is relatively high. A more detailed analysis revealed a preference for physical robots over digital avatars. Also, highly anthropomorphic robot avatars were the most favored appearance type, followed by cartoonish and android designs. Hybrid androids and animal-like robots were less accepted, indicating discomfort with ambiguous or zoomorphic forms. Acceptance also varied significantly by context: commercial, transit, and cultural environments were considered appropriate venues for robot deployment, while schools and healthcare settings received much lower levels of support. These findings suggest that citizens are comfortable with robotic systems for customer service but might be more reluctant to adopt them in spaces that involve more vulnerable populations such as kids, patients, or older adults.

Functional roles also influenced acceptance. Participants expressed strong support for service-oriented and informational tasks, such as guidance and multilingual assistance, but showed greater hesitation toward emotionally sensitive functions like companionship or handling complaints. This pattern reinforces the importance of matching avatar functionality with perceived social appropriateness.

Demographic analyses highlighted that cultural background shapes attitudes toward avatar appearance and modality. Emiratis exhibited higher acceptance of both physical and digital avatar formats, while South Asians were less accepting of these modalities. The results also indicated that acceptance of robot avatar appearances varies markedly across community clusters, with only the highly anthropomorphic robotic-looking design showing comparable acceptance across groups. The most pronounced differences in appearance preferences emerged for android and cartoonish robot designs. Emirati respondents showed notably higher acceptance of android avatars, whereas participants from the ‘Other Asia’ cluster were markedly less accepting of this appearance type. At the same time, ‘Other Asia’ respondents displayed a strong preference for cartoonish robot designs, in contrast to Western participants, who showed much lower acceptance for cartoonish appearances. Together, these patterns indicate that sociocultural and geographic background meaningfully shapes appearance preferences for robot avatars.

Gender analyses revealed that men were significantly more accepting of androids, while no other appearance or modality showed gender-based differences.

These findings carry practical implications for the development and deployment of cybernetic systems. Adaptability in visual style may help align robots with the preferences of specific communities. Moreover, deployment strategies should consider the function and location of robots, ensuring that they are introduced in contexts where they are likely to be perceived as beneficial and appropriate and be particularly mindful when they are intended for interactions in spaces that involve vulnerable populations.

Several limitations should be acknowledged. First, the study relied on still images to represent avatar appearances, which may not fully capture the dynamic and behavioral qualities that influence real-world acceptance. Second, the survey context was hypothetical, asking participants to imagine future scenarios rather than responding to direct interaction. In the future, real exposure to robots may lead to different results. In this regard, it would be particularly valuable to conduct a longitudinal follow-up to examine how public attitudes evolve over time as individuals gain firsthand experience with these systems in real-life settings.

Our study offers a unique contribution to the field by capturing perspectives from residents of over 80 countries. To our knowledge, no previous work in the domain of human–robot interaction has addressed the topic of avatar acceptance with a similar breadth of cultural representation. The multicultural structure of Dubai allowed us to gather views from highly diverse backgrounds within a single urban setting, enabling a comparative lens that is rarely accessible in conventional population samples. This cross-cultural richness positions the present dataset as an empirical foundation for future research seeking to generalize findings across global contexts.

As society increasingly considers integrating robotic and virtual agents into service infrastructures, understanding public sentiment is essential. By identifying where and how different formats and appearances are accepted—or resisted—this research contributes actionable insights for inclusive and culturally sensitive deployment. Importantly, the implications of this research extend beyond survey data collection. This study has been supported also as part of a concrete strategy to guide real-world deployments. The insights gained are intended to directly inform the future design and implementation of cybernetic avatars—both robotic and digital—across public and private service sectors in the country. In this sense, the project exemplifies science-driven innovation: rigorous empirical research serving as the foundation for immediate societal impact.

While this study centers on cybernetic avatars, the findings are also relevant to the domains of social and humanoid robotics, which might trigger similar acceptance as robot avatars, particularly concerning appearance, settings, and tasks. As such, future research could also expand on our findings to refine the conceptual boundaries of the “uncanny valley” in multicultural contexts.

As robots become increasingly integrated into various aspects of daily life, understanding public acceptance of cybernetic avatars and social robots in general is crucial for a successful deployment. Incorporating citizen feedback into the development and deployment of these technologies throughout all stages is critical to ensure a harmonious coexistence between humans and robots in our future society.

## Data Availability

The raw data supporting the conclusions of this article will be made available by the authors, without undue reservation.

## Footnotes

1 During their participation, the participants completed additional blocks of questions related to acceptance of robot avatars for capability enhancement and the design of an ideal robot avatar for customer service which are not reported here.
