# Supplementary material for: Public acceptance of cybernetic avatars in the service sector: evidence from a large-scale survey
Source: Front Robot AI. 2026 Jan 12;12:1719342. doi: 10.3389/frobt.2025.1719342 (PMC12832308; doi:10.3389/frobt.2025.1719342)
Supplement: Supplementary file 1 [file Supplementaryfile1.docx]

**SUPPLEMENTARY INFORMATION**

**SURVEY QUESTIONS**

**INTRODUCTION TO ROBOT AVATARS**

**Robot avatars** are hybrid interaction robots that combine autonomous capabilities with teleoperated control. They can interact socially and perform tasks on their own, while also being controlled by a human operator for real-time interaction.


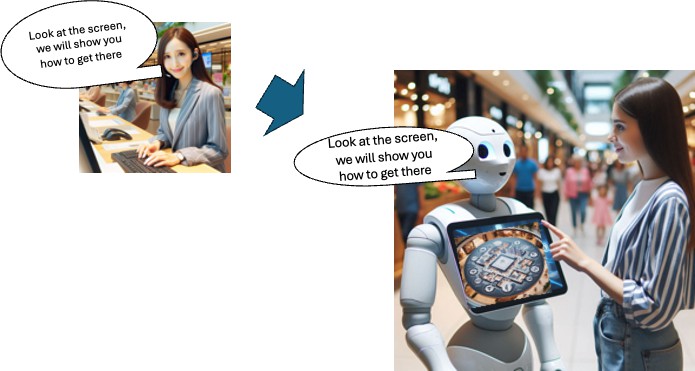


Below, you can see an example of a real robot avatar being teleoperated


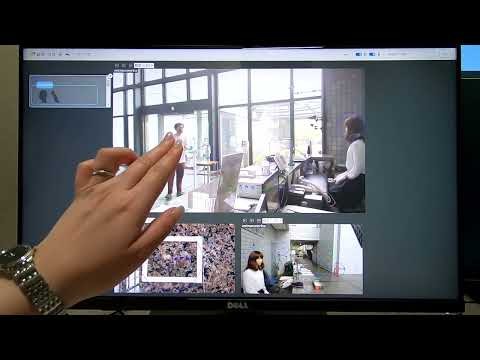
 [http://youtube.com/watch?](http://youtube.com/watch?v=EHS498bg8HM) [v=EHS498bg8HM](http://youtube.com/watch?v=EHS498bg8HM)

**ROBOT AVATARS FOR THE SERVICE SECTOR**

**Imagine robot avatars are introduced in Dubai to assist customers in spaces such as shopping malls, governmental oﬃces, museums, hotel lobbies, conference centers, banks, metro stations, or the airport.**

**APPEARANCE**

**In the ideal society you envision for Dubai, robot avatars designed to assist customers in the service sector should have the following appearances:**

[disagree – neutral – agree]

*(Note. The images are just examples; please focus on the broader category they represent)*

1. **ULTRA-REALISTIC ANDROIDS**

Robots that have appearances very similar to a human being, with skin-looking texture


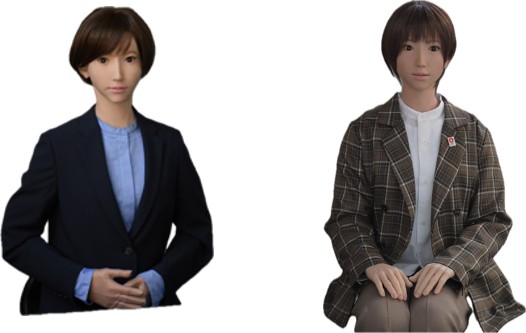


1. **HYBRID ANDROIDS**

Robots that are a mix of human and robotic appearance


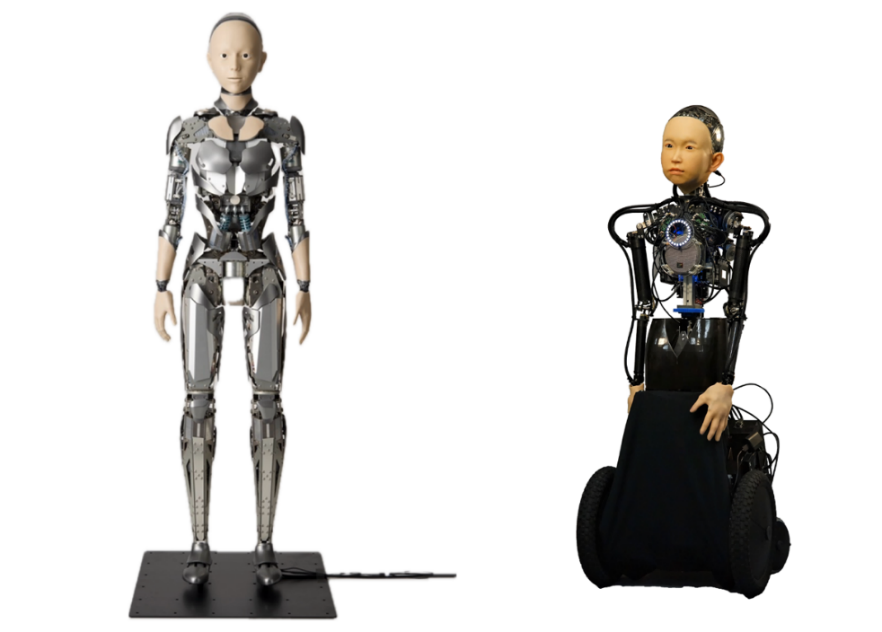


1. **ROBOTIC-LOOKING, HIGHLY ANTHROPOMORPHIC**

Robots that have body shapes and faces that remind humans but clearly look robotic


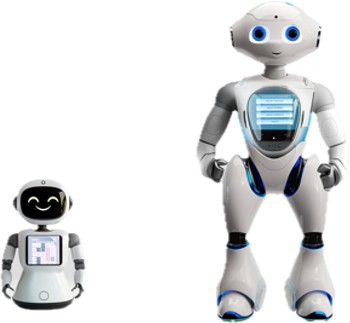


1. **ROBOTIC-LOOKING, LOW ANTHROPOMORPHIC**

Robots that have minimal human-like features


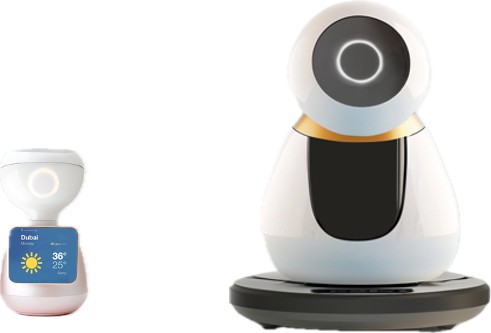


1. **CARTOONISH-LOOKING**

Robots that resemble animated characters
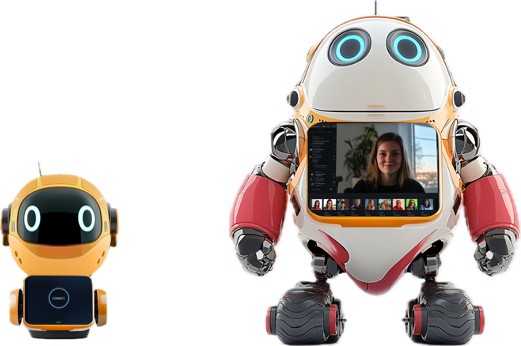


1. **ANIMAL-LOOKING**

Robots that mimic the appearance of animals


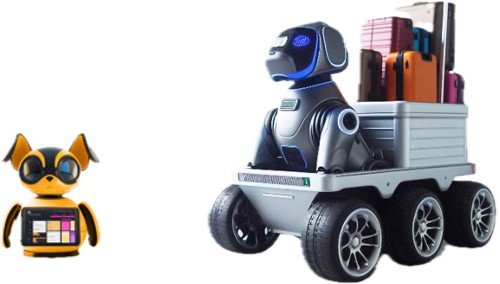


**UNDERSTANDING ANDROID ACCEPTANCE / REJECTION**

**We are particularly interested in understanding the reasons behind your choice regarding ultra-realistic androids (i.e., robots with human-like appearances, including skin-like textures). You selected '<ANSWER> about deploying these specific robots for customer service in Dubai because...**

**SPACES**

**In the ideal society you envision for Dubai, robot avatars designed to assist customers in the service sector should be allowed in the following spaces:**

[disagree – neutral – agree]

Hospitals and clinics

Dental clinics

Pharmacies

Rehabilitation centres

Nursing homes

Schools and High Schools

Language academies

Universities

Banks

Stores

Supermarkets

Shopping malls

Government offices

Libraries

Post Offices

Police stations

Museums

Airports

Train and Metro stations

Hotels

Restaurants, bars and coffee shops

Beach clubs

Conference centres

Public spaces such as parks or streets

**TASKS**

**In the ideal future society you envision for Dubai, robot avatars designed to assist customers in the service sector should perform the following tasks:**

[disagree – neutral – agree]

Provide information to customers

Customer Registration / Check in – out

Provide indications and guidance to find a place

Telepresence to speak with a customer service representative

Patrolling spaces for security

Companionship and entertainment functions

Object delivery (e.g. documents, packages, shopping bags)

Customer feedback and surveys

Handle customer complaints

Sign up to loyalty programs

Collect used batteries, light, bulbs, glass, and old clothes for recycling

Carry your shopping bags or suitcase

Multilingual support

I am paying attention to the survey, select “Agree”

**MODALITY**

**In the ideal society you envision for Dubai, avatars that are designed to assist customers in the service sector should exist in the following modalities:**

[disagree – neutral – agree]


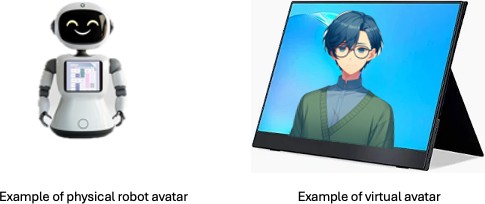


Physical in the form of robots

Digital in virtual reality or screens

**RELATIONSHIP WITH ROBOTS AND TECHNOLOGY**

**Indicate your general level of interest in scientific discoveries and technological developments**

Not interested

Moderately interested

Very interested

**Generally speaking, do you have a view of robots**

Very negative

Fairly negative

Fairly positive

Very positive

**Overall, how afraid are you of robots?**

Not at all

Slightly

Moderately

Very

Extremely

**DEMOGRAPHICS**

**How long have you lived in Dubai?**

Less than one year

1 - 3 years

4 - 10 years

More than 10 years

**Were you born in the UAE?**

Yes

No

**Do you have a background in Computer Science, Engineering, or Robotics?**

Yes

No

**Please indicate your level of programming skills.**

None

Basic

Medium

Advanced

**Have you ever used or are you currently using robots.**

At home

At work

Other places (e.g. shopping mall, airport, …)

**Please indicate your experience interacting with robots.**

I have never interacted with robots

I have occasionally interacted with robots

I have ample experience interacting with robots

**Please indicate your experience interacting with robot avatars.**

I have never interacted with robot avatars

I have occasionally interacted with robot avatars

I have ample experience interacting with robot avatars

**What religious family do you identify yourself most close to?**

Islam

Hinduism

Christianity

Buddhism

Judaism

Irreligion

I'd rather not answer

Other (please specify): ______________

**To what extent do you consider yourself to be religious?**

Not at all ----- Very much so

**For each statement, please rate the extent to which you believe it describes you:**

I'd rather depend on myself than others

If a coworker gets a prize, I would feel proud

It is important that I do my job better than others

Parents and children must stay together as much as possible

**Is there anything else you would like to share with us regarding robot avatars for customer service in Dubai or about this survey?**
