# Supplementary material for: Public acceptance of cybernetic avatars in the service sector: evidence from a large-scale survey
Source: Front Robot AI. 2026 Jan 12;12:1719342. doi: 10.3389/frobt.2025.1719342 (PMC12832308; doi:10.3389/frobt.2025.1719342)
Supplement: Supplementary file 2 [file Supplementaryfile2.docx]

**SUPPLEMENTARY INFORMATION**

**TABLES**

Table 1A

Significant Deviations in Acceptance of Physical Robot Avatars by Community Cluster

|  | Disagree | Neutral | Agree | Interpretation |
| --- | --- | --- | --- | --- |
| Emirati |  |  | 2.1 | Higher Agreement |
| Middle East |  |  |  |  |
| South Asia | 2.8 |  |  | Higher Disagreement |
| Other Asia |  |  |  |  |
| Western |  |  |  |  |
| Other Africa |  |  |  |  |

*Note. Values represent Adjusted Standardized Residuals (ASRs). Only cells with |ASR| ≥ 2.0 (approx.* p *< .05) are shown.*

Table 2A

Significant Deviations in Acceptance of Digital Robot Avatars by Community Cluster

|  | Disagree | Neutral | Agree | Interpretation |
| --- | --- | --- | --- | --- |
| Emirati |  |  | 2.2 | Higher Agreement |
| Middle East |  |  |  |  |
| South Asia | 2.7 |  | -2.6 | Higher Disagreement, Lower Agreement |
| Other Asia |  |  |  |  |
| Western |  |  |  |  |
| Other Africa |  |  |  |  |

*Note. Values represent Adjusted Standardized Residuals (ASRs). Only cells with |ASR| ≥ 2.0 (approx.* p *< .05) are shown.*

*Table 3A*

Significant Deviations in Acceptance of Android Avatars by Community Cluster

|  | Disagree | Neutral | Agree | Interpretation |
| --- | --- | --- | --- | --- |
| Emirati | -2.3 | -4.0 | 5.4 | Higher Agreement, Lower Disagreement, Fewer Neutral |
| Middle East |  |  |  |  |
| South Asia |  |  |  |  |
| Other Asia |  | 5.2 | -5.0 | Lower Agreement, Higher Neutrality |
| Western | 2.7 | -2.1 |  | Higher Disagreement, Fewer Neutrality |
| Other Africa |  |  |  |  |

*Note. Values represent Adjusted Standardized Residuals (ASRs). Only cells with |ASR| ≥ 2.0 (approx.* p *< .05) are shown.*

*Table 4A*

Significant Deviations in Acceptance of Hybrid Android Avatars by Community Cluster

|  | Disagree | Neutral | Agree | Interpretation |
| --- | --- | --- | --- | --- |
| Emirati | -2.3 |  |  | Lower Disagreement |
| Middle East |  |  |  |  |
| South Asia |  |  |  |  |
| Other Asia |  | 3.1 | -2.8 | Lower Agreement, Higher Neutrality |
| Western | 2.0 |  |  | Higher Disagreement |
| Other Africa |  |  |  |  |

*Note. Values represent Adjusted Standardized Residuals (ASRs). Only cells with |ASR| ≥ 2.0 (approx.* p *< .05) are shown.*

*Table 5A*

Significant Deviations in Acceptance of Robotic-Looking, Low Anthropomorphic Avatars by Community Cluster

|  | Disagree | Neutral | Agree | Interpretation |
| --- | --- | --- | --- | --- |
| Emirati | -4.0 |  | 2.2 | Higher Agreement, Lower Disagreement |
| Middle East |  |  |  |  |
| South Asia |  |  | -2.3 | Lower Agreement |
| Other Asia | -3.3 |  |  | Lower Disagreement |
| Western |  |  |  |  |
| Other Africa | 2.4 |  |  | Higher Disagreement |

*Note. Values represent Adjusted Standardized Residuals (ASRs). Only cells with |ASR| ≥ 2.0 (approx.* p *< .05) are shown.*

*Table 6A*

Significant Deviations in Acceptance of Cartoonish Avatars by Community Cluster

|  | Disagree | Neutral | Agree | Interpretation |
| --- | --- | --- | --- | --- |
| Emirati |  |  |  |  |
| Middle East |  |  |  |  |
| South Asia | 2.3 |  |  | Higher Disagreement |
| Other Asia | -4.1 |  | 4.4 | Higher Agreement, Lower Disagreement |
| Western | 2.6 |  | -3.6 | Higher Disagreement, Lower Agreement |
| Other Africa |  |  |  |  |

*Note. Values represent Adjusted Standardized Residuals (ASRs). Only cells with |ASR| ≥ 2.0 (approx.* p *< .05) are shown.*

*Table 7A*

Significant Deviations in Acceptance of Animal-looking Avatars by Community Cluster

|  | Disagree | Neutral | Agree | Interpretation |
| --- | --- | --- | --- | --- |
| Emirati |  |  | 2.2 | Higher Agreement |
| Middle East |  |  | -2.0 | Lower Agreement |
| South Asia |  |  |  |  |
| Other Asia | -3.1 |  |  | Lower Disagreement |
| Western |  |  | -2.1 | Lower Agreement |
| Other Africa |  | -2.2 |  | Fewer Neutral |

*Note. Values represent Adjusted Standardized Residuals (ASRs). Only cells with |ASR| ≥ 2.0 (approx.* p *< .05) are shown.*

*Note: Non-significant results were found for Robotic-Looking, High Anthropomorphic avatars – further analyses not performed*
